# Supplementary material for: Is educational attainment associated with the onset and outcomes of low back pain? a systematic review and meta-analysis
Source: PLoS One. 2024 Aug 13;19(8):e0308625. doi: 10.1371/journal.pone.0308625 (PMC11321572; doi:10.1371/journal.pone.0308625)
Supplement: S1 Appendix — (DOCX) [file pone.0308625.s002.docx]

**Search Strategy**

exp Socioeconomic factors/ or exp sociodemographic factors/ or exp "social determinants of health"/ or exp educational status/ or exp social class/

((educat* or academic) adj5 (attainment or level or status or achieve* or formal or higher or college* or universit*)).ti,ab,kw.

1 or 2

exp back pain/ or exp low back pain/

(back pain or low back pain or lumbago or low backache).ti,ab,kw.

((pain or complaint or disability or disorder) adj5 (back or low* back or lumbar or lumbar spine or dorsalgia or backache)).ti,ab,kw.

4 or 5 or 6

(cohort or population or prospective or retrospective or "prognos*" or incidence or "predict*" or survival or "causal factor" or risk or course).ti,ab,kw,tw.

exp Prognosis/ or exp Cohort Studies/ or exp Risk/ or exp incidence/ or exp survival analysis/

8 or 9

3 and 7 and 10
