# Supplementary material for: Is educational attainment associated with the onset and outcomes of low back pain? a systematic review and meta-analysis
Source: PLoS One. 2024 Aug 13;19(8):e0308625. doi: 10.1371/journal.pone.0308625 (PMC11321572; doi:10.1371/journal.pone.0308625)
Supplement: Attachment — Submitted filename: COMENT-CARLOS-REVIEWER-PONE-D-24-08660_reviewer.pdf [file pone.0308625.s003.pdf]

PLOS ONE

Is educational attainment associated with the onset and outcomes of low back pain? A systematic review and meta-analysis

--Manuscript Draft--

Manuscript Number: PONE-D-24-08660

Article Type: Research Article

Full Title: Is educational attainment associated with the onset and outcomes of low back pain? A systematic review and meta-analysis

Short Title: educational attainment and the onset and outcomes of low back pain

Corresponding Author: Aliyu Lawan, PhD
University of Western Ontario: Western University
London, Ontario CANADA

Keywords: low back pain; educational attainment; low back pain disability; back pain outcomes; prognosis

Abstract:

Background: Low back pain (LBP) is the leading global cause of years lived with disability. Of the biopsychosocial domains of health, social determinants of LBP remain under-researched. Socioeconomic status (SES) may be associated with the onset of new LBP or outcomes of acute LBP, with educational attainment (EA) being a key component of SES. The association between EA and LBP has yet to be the subject of a dedicated review and meta-analysis.
Purpose: To review evidence of the association between EA and a) onset or b) outcomes of acute and subacute LBP in the adult general population and to conduct statistical pooling of data where possible.
Methods: An electronic search was conducted in MEDLINE, Embase, CINAHL, and ProQuest from inception to November 2023 including reference lists to identify relevant prospective studies. Risk of bias (RoB) was assessed using the Quality in Prognostic Studies (QUIPS) tool. Where adequate data were available, estimates were pooled using a random-effects meta-analysis. Overall evidence for each outcome was graded using an adapted GRADE.
Results: After screening 8498 studies, 29 were included in the review. Study confounding and attrition were common biases. Data from 21 studies were statistically pooled to explore EA as a predictor of new LBP onset or as prognostic for outcomes of acute or subacute LBP. Pooled results showed no association between EA and the onset of new LBP (OR: 0.927, 95%CI: 0.747 to 1.150; I2= 0%). For predicting outcomes of acute LBP, compared to those with no more than secondary -level education, post-secondary education or higher was associated with better outcomes of pain (OR: 0.538, 95%CI: 0.432 to 0.671; I2= 35%) or disability (OR: 0.565, 95%CI: 0.420 to 0.759; I2= 44%). High heterogeneity (I2>80%) prevented meaningful pooling of estimates for subacute LBP outcomes.
Conclusion: We found no consistent evidence that lower EA increases risk of LBP onset. Lower EA shows consistent association with worse LBP outcomes measured at least 3 months later after acute onset with inconclusive findings in subacute LBP. Causation cannot be supported owing to study designs. Discussion is needed for potential mechanisms to explain these effects, though high-quality research focusing on social determinants of LBP is needed.

Order of Authors:

Aliyu Lawan, PhD
Alex Aubertin
Jane Mical
Joanne Hum
Michelle L. Graf
Peter Marley
Zachary Bolton
David M. Walton

Powered by Editorial Manager® and ProduXion Manager® from Aries Systems Corporation

Additional Information:	
**Question**	**Response**
**Financial Disclosure**<br><br>Enter a financial disclosure statement that describes the sources of funding for the work included in this submission. Review the submission guidelines for detailed requirements. View published research articles from *PLOS ONE* for specific examples.<br><br>This statement is required for submission and **will appear in the published article** if the submission is accepted. Please make sure it is accurate.<br><br>**Funded studies**<br>Enter a statement with the following details:<br>• Initials of the authors who received each award<br>• Grant numbers awarded to each author<br>• The full name of each funder<br>• URL of each funder website<br>• Did the sponsors or funders play any role in the study design, data collection and analysis, decision to publish, or preparation of the manuscript?<br><br>Did you receive funding for this work?	The author(s) received no specific funding for this work.
**Competing Interests**<br><br>Use the instructions below to enter a competing interest statement for this submission. On behalf of all authors, disclose any competing interests that could be perceived to bias this work—acknowledging all financial support and any other relevant financial or non-financial competing interests.<br><br>This statement is required for submission and **will appear in the published article** if the submission is accepted. Please make sure it is accurate and that any funding sources listed in your Funding Information	The authors have declared that no competing interests exist.

later in the submission form are also declared in your Financial Disclosure statement.<br><br>View published research articles from *PLOS ONE* for specific examples.<br><br>**NO authors have competing interests**<br><br>Enter: *The authors have declared that no competing interests exist.*<br><br>**Authors with competing interests**<br><br>Enter competing interest details beginning with this statement:<br><br>*I have read the journal's policy and the authors of this manuscript have the following competing interests: [insert competing interests here]*<br><br>* typeset	
**Ethics Statement**<br><br>Enter an ethics statement for this submission. This statement is required if the study involved:<br><br>• Human participants<br>• Human specimens or tissue<br>• Vertebrate animals or cephalopods<br>• Vertebrate embryos or tissues<br>• Field research<br><br>Write "N/A" if the submission does not require an ethics statement.<br><br>General guidance is provided below. Consult the submission guidelines for detailed instructions. **Make sure that all information entered here is included in the Methods section of the manuscript.**	No ethics approval is required for this study.

**Format for specific study types**<br><br>**Human Subject Research (involving human participants and/or tissue)**<br>• Give the name of the institutional review board or ethics committee that approved the study<br>• Include the approval number and/or a statement indicating approval of this research<br>• Indicate the form of consent obtained (written/oral) or the reason that consent was not obtained (e.g. the data were analyzed anonymously)<br><br>**Animal Research (involving vertebrate animals, embryos or tissues)**<br>• Provide the name of the Institutional Animal Care and Use Committee (IACUC) or other relevant ethics board that reviewed the study protocol, and indicate whether they approved this research or granted a formal waiver of ethical approval<br>• Include an approval number if one was obtained<br>• If the study involved *non-human primates*, add *additional details* about animal welfare and steps taken to ameliorate suffering<br>• If anesthesia, euthanasia, or any kind of animal sacrifice is part of the study, include briefly which substances and/or methods were applied<br><br>**Field Research**<br><br>Include the following details if this study involves the collection of plant, animal, or other materials from a natural setting:<br>• Field permit number<br>• Name of the institution or relevant body that granted permission	
**Data Availability**<br><br>Authors are required to make all data underlying the findings described fully available, without restriction, and from the time of publication. PLOS allows rare exceptions to address legal and ethical concerns. See the PLOS Data Policy and FAQ for detailed information.	Yes - all data are fully available without restriction

A Data Availability Statement describing where the data can be found is required at submission. Your answers to this question constitute the Data Availability Statement and **will be published in the article**, if accepted.<br><br>**Important:** Stating 'data available on request from the author' is not sufficient. If your data are only available upon request, select 'No' for the first question and explain your exceptional situation in the text box.<br><br>Do the authors confirm that all data underlying the findings described in their manuscript are fully available without restriction?	
**Describe where the data may be found in full sentences. If you are copying our sample text, replace any instances of XXX with the appropriate details.**<br><br>• If the data are **held or will be held in a public repository**, include URLs, accession numbers or DOIs. If this information will only be available after acceptance, indicate this by ticking the box below. For example: *All XXX files are available from the XXX database (accession number(s) XXX, XXX.*).<br>• If the data are all contained **within the manuscript and/or Supporting Information files**, enter the following: *All relevant data are within the manuscript and its Supporting Information files.*<br>• If neither of these applies but you are able to provide **details of access elsewhere**, with or without limitations, please do so. For example:<br><br>*Data cannot be shared publicly because of [XXX]. Data are available from the XXX Institutional Data Access / Ethics Committee (contact via XXX) for researchers who meet the criteria for access to confidential data.*<br><br>*The data underlying the results presented in the study are available from (include the name of the third party*	The data underlying the results presented in the study are available within the manuscript.

and contact information or URL).<br>• This text is appropriate if the data are owned by a third party and authors do not have permission to share the data.<br><br>* typeset	
Additional data availability information:	

Manuscript
Click here to access/download;Manuscript;Manuscript.docx

Is educational attainment associated with the onset and outcomes of low back pain? A systematic review and meta-analysis

Aliyu Lawan[1], Alex Aubertin[2], Jane Mical[1], Joanne Hum[3], Michelle L. Graf[4], Peter Marley[1], Zachary Bolton[1], David M. Walton[1]

1: Faculty of Health Sciences, Western University, London ON
2: School of Health Sciences, Nursing, and Emergency Services, Cambrian College, Sudbury, ON
3: Fraser Health Authority, New Westminster, BC
4: Department of Physical Therapy, University of British Columbia, Vancouver, BC

Corresponding Author:
David M. Walton, BScPT, MSc, PhD
Associate Professor, School of Physical Therapy
Rm. 2300A, Western University
1201 Western Rd. London, ON
N6G 1H1

Data Availability Statement: The raw data involved in this analysis are available within the manuscript.

Disclosures: No funding was received for this project. The authors declare no conflict of interest with the nature of the work described.

Running title: educational attainment and the onset and outcomes of low back pain

Author Contributions
Conceptualization: David M. Walton
Data curation: Aliyu Lawan, Alex Aubertin, Jane Mical, Joanne Hum, Michelle L. Graf, Peter Marley, Zachary Bolton, David M. Walton
Formal analysis: Aliyu Lawan, Alex Aubertin, Jane Mical, Joanne Hum, Michelle L. Graf, Peter Marley, Zachary Bolton, David M. Walton
Investigation: Aliyu Lawan and David M. Walton
Methodology: David M. Walton
Project administration: David M. Walton
Resources: David M. Walton
Validation: David M. Walton
Writing – original draft: Aliyu Lawan, Alex Aubertin, Jane Mical, Joanne Hum, Michelle L. Graf, Peter Marley, Zachary Bolton, David M. Walton
Writing – review & editing: David M. Walton

Abstract

Background: Low back pain (LBP) is the leading global cause of years lived with disability. Of the biopsychosocial domains of health, social determinants of LBP remain under-researched. Socioeconomic status (SES) may be associated with the onset of new LBP or outcomes of acute LBP, with educational attainment (EA) being a key component of SES. The association between EA and LBP has yet to be the subject of a dedicated review and meta-analysis.

Purpose: To review evidence of the association between EA and a) onset or b) outcomes of acute and subacute LBP in the adult general population and to conduct statistical pooling of data where possible.

Methods: An electronic search was conducted in MEDLINE, Embase, CINAHL, and ProQuest from inception to 2nd November 2023 including reference lists to identify relevant prospective studies. Risk of bias (RoB) was assessed using the Quality in Prognostic Studies (QUIPS) tool. Where adequate data were available, estimates were pooled using a random-effects meta-analysis. Overall evidence for each outcome was graded using an adapted GRADE.

Results: After screening 8498 studies, 29 were included in the review. Study confounding and attrition were common biases. Data from 21 studies were statistically pooled to explore EA as a predictor of new LBP onset or as prognostic for outcomes of acute or subacute LBP. Pooled results showed no association between EA and the onset of new LBP (OR: 0.927, 95%CI: 0.747 to 1.150; I^2= 0%). For predicting outcomes of acute LBP, compared to those with no more than secondary -level education, post-secondary education or higher was associated with better outcomes of pain (OR: 0.538, 95%CI: 0.432 to 0.671; I^2= 35%) or disability (OR: 0.565, 95%CI: 0.420 to 0.759; I^2= 44%). High heterogeneity (I^2>80%) prevented meaningful pooling of estimates for subacute LBP outcomes.

Conclusion: We found no consistent evidence that lower EA increases risk of LBP onset. Lower EA shows consistent association with worse LBP outcomes measured at least 3 months later after acute onset with inconclusive findings in subacute LBP. Causation cannot be supported owing to study designs. Discussion is needed on potential mechanisms to explain these effects, though high-quality research focusing on social determinants of LBP is needed.

Keywords: low back pain, educational attainment, low back pain disability, back pain outcomes, prognosis

INTRODUCTION

Low back pain (LBP) is a leading global cause of years lived with disability.[1] In North America, chronic LBP is amongst the top ten reasons for seeking medical attention[2] with a prevalence of 18-23% in adults Canadians.[3] While many LBP cases resolve within the first three months, it has been estimated that as many as 60% to 80% progresses to chronicity or recurrence within one year including loss of productivity in 40%.[4,5] The acute to chronic transition of LBP is a complex process with multiple mechanisms likely influencing the pathway including biological, psychological, and social.[6–8] Prevention of new LBP or prevention of the acute-to-chronic transition stand to have major impact on global health burden.[2] Existing guidelines recommend early identification of psychosocial factors that could prevent or enhance recovery from LBP.[9]

While the biological and psychological sciences have provided considerable evidence to explain onset of and recovery from LBP, much less attention has been paid to the social influences. Social determinants of health (SDOH) are increasingly recognized as potent influences on the genesis of several health states,[10] with some prior authors indicating that neighborhood characteristics may have at least as large an influence on the experience of chronic diseases as do personal genetics.[11] While SDOHs represent a large and complicated field of research, there are some social variables unique to the person experiencing pain that are worthy of dedicated inquiry. One such variable is educational attainment (EA), defined as the highest level of education completed by a person. As a prognostic variable, EA represents a blend of person-level (e.g., literacy) and society-level (e.g., access) influences and could potentially hold value as a variable through which intervention strategies could be tailored. EA holds value for research on SDOHs as "years of education" is one of few such variables that can be readily quantified.

There is some evidence that chronic pain is more prevalent amongst people with low EA[12] and lower EA may predict the acute-to-chronic transition.[13] However, there are limited studies that focus solely on the social predictors of LBP specifically.[6] EA has been included in some prior systematic reviews in LBP[14,15] though differences in case definitions, variable definitions, or study design have precluded clear findings. Even rarer are reviews or evidence syntheses on the association between EA and the onset of new LBP in population-level cohort studies that start with pain-free participants.[16] If lower EA is a risk for new onset LBP or for poor recovery following onset of acute LBP, mechanisms could then be explored and if causation is supported EA could be integrated into either public health prevention strategies or tailored treatment planning to prevent the acute-to-chronic transition.

The purpose of this systematic review was to qualitatively and/or quantitatively synthesize published estimates on the risk and prognostic value of EA on the onset or outcomes of LBP.

METHODS

Design

This review was designed and reported according to the Preferred Reporting Items for Systematic Reviews and Meta-Analyses (PRISMA) framework. The review was limited to observational prospective cohort or population-level studies (not clinical trials) of patients aged ≥18 years with either no LBP at inception (followed to determine onset), or acute (<8 weeks) and/or sub-acute (8-12 weeks) LBP. We focused on 'non-specific' LBP and therefore excluded LBP related to underlying systemic medical (such as cancer, infection, or cauda equina syndrome), vertebral compression fracture or osteoporosis, inflammatory conditions (e.g., ankylosing spondylitis) or neurological conditions (e.g., stroke). Beyond that, we accepted the case definitions of LBP as reported by the authors of the primary sources.

Search Strategy

The search strategy (Appendix A) was developed with a research librarian using MeSH terms specific to MEDLINE which was adapted for other databases. No specific restrictions on publication date were set. The search strategies were applied to MEDLINE (OVID), The Cumulative Index of Nursing and Allied Health Literature (CINAHL), EMBASE, and ProQuest from inception to 30th March 2023 and updated to 2nd November 2023 corresponding to the dates of the respective searches. A grey literature search of unpublished studies was conducted in Researchsquare. Hand searches of reference lists of all included articles were conducted to identify additional primary sources.

Study Selection

Yield from each database were imported into Covidence systematic review software (Veritas Health Innovation, Melbourne Australia) and screened by two independent reviewers against the inclusion criteria, with disagreements being resolved by a third reviewer. Titles and abstracts were screened to remove clearly irrelevant sources, followed by full text screening against the inclusion/exclusion criteria. Kappa was calculated as an indicator of agreement between raters. The reasons for exclusion are included in Figure 1.

Risk of bias in individual studies

The Quality in Prognostic Studies (QUIPS) tool was used to assess RoB of all included studies. QUIPS consists of six category-domains of potential biases: i) study participation, ii) attrition, iii) prognostic factor measurement, iv) outcome measurement, v) confounding, and vi) statistical analysis / reporting. All included studies were assessed by 2 independent reviewers. We used a worst-score approach, where each paper was assigned a RoB based on the worst (highest risk) rating of any of the 6 categories[17] classed as low, moderate, or high risk of bias

(RoB). RoB agreement was calculated through Cohen's kappa with disagreements resolved through discussion with a third experienced reviewer.

Data Extraction

Data were extracted using a study-specific extraction table that included key study descriptors, sample characteristics, operationalizations of EA and LBP, outcomes assessed, and relevant findings. Educational attainment was extracted with as much detail as reported in the publication. Where possible, the minimum data extracted were related to a 12-year cut-point for EA as representing the threshold between secondary (up to year 12) and post-secondary (beyond year 12) EA in most countries. Where data were not presented with adequate detail, EA was sorted into meaningful order based on the manner reported in the studies (e.g., low vs high). We did not restrict studies based on the length of follow-up but extracted that information for subsequent interpretation as a potential effect modifier.

Outcomes were limited to those broadly categorized as either pain (e.g., presence/absence of LBP or pain intensity) or disability (e.g., return to work or score on a standardized patient-reported outcome). Where studies reported "recovery" as an outcome those operationalizations were reviewed for relevance and if aligned with our purpose the verbatim definition was extracted and assigned to the most relevant outcome category (e.g., pain, disability, or both). The study protocol was prospectively registered in PROSPERO (registration no. CRD 42023402135) as part of a series of reviews on SDOHs and LBP.

Data analysis and synthesis

Where possible, data were pooled and presented as odd ratio through random-effects meta-analysis using Comprehensive Meta-analysis software, version 2.2.04 (Biostat, Inc.©,

Englewood, New Jersey). Syntheses were conducted for each of: i) onset of LBP (inception cohorts that start with no LBP and are followed over time to identify those who later report LBP); ii) pain intensity outcomes in acute LBP (inception starting within 8 weeks of LBP onset and followed over time to evaluate recovery), iii) pain-related disability outcomes in acute LBP, and iv) pain or disability outcomes in those entering the study with subacute (8 to 12 weeks) LBP. Heterogeneity in effects was assessed using both the Chi-squared test (p-value) and the I-squared statistic. I-squared <30% was deemed low heterogeneity, 30-60% as moderate, 61-75% as substantial, and 76-100% as considerable heterogeneity.[18] First, one estimates for pain and/or disability were pooled for unadjusted from each study and where the unadjusted is not available, the adjusted is pooled. Where substantial or considerable heterogeneity was identified, and where enough primary sources were available, sensitivity analysis was undertaken to explore potential sources of heterogeneity by pooling only the unadjusted estimates in each study.

When heterogeneity could not be explained, or where there were too few primary sources to permit moderator analysis in otherwise highly heterogeneous effects, a narrative summary of the results is presented.

GRADE assessment

Results across studies were synthesized using a modified Grading of Recommendations Assessment, Development and Evaluation (GRADE) approach that considered the strength of the effect (none, small, medium, or high) and confidence in the results (inconclusive, low, moderate, or high) based on RoB, precision, homogeneity and consistency of effects. Where effects could be statistically pooled, those results were used to determine effect size, where they could not, we used a qualitative synthesis approach focused on overall consistency across

papers. For this review, we did not attempt to find study registration through online registries to identify publication bias as observational studies are not consistently registered and many studies were published prior to protocol registration becoming standard practice.

RESULTS

Figure 1 shows the PRISMA flow diagram. The search identified 8498 articles (including 1058 duplicates), of which 163 full texts were screened resulting in the inclusion of 23 articles. An additional 4 from reference lists and 2 from the update search were identified for a total of 29 manuscripts describing 27 prospective observational cohorts. The average agreement between raters was Kappa = 0.54, and random agreement was 0.97. Characteristics of the included studies are summarized in Table 1. The included studies were grouped into: onset of new LBP (n=3), outcome of acute LBP (n=18) and outcome of subacute LBP (n=8). The publication date of the included studies spanned 1991 to 2022 and were from 13 countries. Sample sizes ranged from 53[19] to 12,500[20] and follow-up periods from 3 months (n = 4[21–24]) to 3 years (n = 2 [20,25]).

Fig. 1: Flowchart of study selection and inclusion.

Risk of Bias

Details of the RoB are reported in Table 1 and RoB for the overall body of literature is presented in Figure 2. The majority (n = 14) of the included studies were rated as high RoB with 13 rated moderate and 2 low. For the individual domains, low RoB was common in the domains of study participation (77%) and statistical analysis/reporting (74%). High RoB was common for the domains of confounding (45%) and study attrition (39%).

Table 1: Summary of findings: Educational attainment predicting the onset and outcomes in acute low back pain.

S/N	Author (year) Country	Aim	Sample and population	Categories of exposure (education)	Categories of outcome (pain or disability)	Design/ follow-up duration	Findings related to education	Study participation	Study attrition	Prognostic factor measurement	Outcome measurement	Confounding	Statistical analysis/reporting	Overall Risk of Bias
**Onset of Back pain**														
1	Zadro[26]<br>2017<br>Australia	Investigate whether EA affects the prevalence and risk of LBP differently in men and women while controlling for the influence of genetics and early shared environment.	1,077 Adult monozygotic (MZ) and dizygotic (DZ) twins from the Murcia Twin Registry	Primary<br>General secondary<br>Superior secondary<br>University	Activity limiting LBP questions: "Was this pain bad enough to limit your usual activities or change your daily routine for more than one day?"	2 to 4 years	EA did not significantly affect the risk of LBP in the combined sample of men and women. Women with general secondary education (OR=0.5, 95% CI: 0.2–0.9, p=.025), and University (adj OR= 0.3, 95% CI: 0.1 – 1.1, p=0.66) have a lower risk of LBP compared to women with primary education.<br><br>EA did not affect the risk of LBP in men.	○	○	⊕	⊕	⊕	○	❂
2	Von Korff[25]<br>1993<br>USA	Assess whether depression was associated with increased risks of onset of 5 anatomically defined pain symptoms in a population-based, prospective study	803 enrollees of a large health maintenance organization, Group Health Cooperative of Puget Sound (GHC).	High school or less<br>Some college<br>College graduate	Back pain: First onset of back pain and in prior 6 months of the bassline	3-year	3-year first onset probabilities of back pain, no significant differences between educational group (p>0.05) in both univariate and multivariate analysis.	○	○	○	○	●	○	✹
3	George[27]<br>2012<br>USA	Identify variables from these domains that were predictive of occurrence of LBP, intensity, disability and psychological distress	1230 consecutive subjects (18–35 years of age, without prior history of back pain) entering a training program at Fort Sam Houston, TX to become a combat medic in the U.S. Army were considered for participation from February 2007 to March 2008.	High school or lower, Some college,<br>College or higher	In this study a prior history of LBP was operationally defined as a previous episode of LBP that limited work or physical activity, lasted longer than 48 hours, and caused the subject to seek health care.	2 years.	High school or lower p=0.204<br>High school or lower versus college or higher OR: 1.013 (0.65 - 1.58), p= 0.954<br>Highest pain intensity ratings for soldiers with high school or lower EA were 1.121 higher (95% CI = [0.239, 2.003]).	○	⊕	●	⊕	●	○	✹
**Acute Back Pain (<6weeks)**														

4	Besen[24]<br>2015<br>USA	Develop and test a model of direct and indirect relationships among individual psychosocial predictors of return-to-work (RTW) outcomes following the onset of LBP.	241 LBP patients with onset of less than 14 days followed from baseline to 3 months.	Education	Return to work	3 months	Education was retained in the model, however for simplicity, we have omitted these paths from which shows the estimated model	⊕	⊕	○	○	○	⊕	✡
5	Grotle[21]<br>2005<br>Norway	examine the clinical course of acute LBP and to evaluate prognostic factors for nonrecovery.	123 patients with acute LBP 3 weeks consulting primary care for the first time were included, and 120 completed 3 months follow-up.	less or more than 12 years	Recovered if they scored ≤ 4 on the RMQ at both the 4 weeks and 3 months follow-up	3 months.	Unadjusted OR=0.59 (0.25–1.38), adjusted OR= 0.68 (0.28–1.69)<br>Adjusting for age and sex	○	○	⊕	○	○	○	✡
6	Grotle[28]<br>2007<br>Norway	Investigate the clinical course of pain and disability, and prognostic factors for non-recovery after 1-year, in patients seeking help for the first time due to acute LBP.	123 (aged 18–60 years) first time acute LBP of <3 weeks duration, with or without radiating pain to the limb, no prior LBP treatment.	<12 years versus ≥12 years	Disability assessed by the RMDQ.<br><br>Non-recovery was defined as an RMQ score of more than 4	1, 3, 6, 9, and 12 months.	High Education compared to low education for non-recovery at 12 months: crude OR= 0.65 (0.24–1.77); Adjusted (Age-and gender) OR: 0.71 (0.26–1.96),<br><br>Adjusted for age and gender	○	●	○	○	●	○	✸
7	Moffett[29]<br>2009<br>UK	Investigate whether socioeconomic status in patients with back pain participating in a randomized controlled trial was predictive of functional disability.	949 secondary analysis was carried out only on UK BEAM (general practices in 14 centers around the UK with nonspecific LBP lasting at least 5 weeks)	Education up to (i) 16 years (or less), (ii) 17–19, (iii) over 20.	Roland Disability Questionnaire	1, 3 and 12 months.	Disability (RDQ scores).<br>Education level ≤16 vs. 17–19: 0.445 (0.21 – 0.942).<br>Education level ≤16 vs. ≥20 0.364 (0.221 – 0.595).<br>At 1, 3, and 12 months respectively.<br>Adjusting for work status and Townsend score	○	⊕	○	⊕	○	○	✡
8	Starkweather[30]<br>2016<br>USA	Examine demographic, pain-related, psychological, and somatosensory characteristics in a cohort of acute LBP with persistent pain or unrecovered after 6 weeks.	106 men and women between the age of 18 and 50 years diagnosed with an acute episode of LBP to participate	High school/ technical or lower; and<br>Start college or higher	Recovered: if their pain resolved in the first 6 weeks after onset or as Persistent: if their pain continued for 6 months	6 months	Persistent: High school=13 (68%) v College=6 (32%)<br><br>Recovered: High school =7 (24%), College= 22 (76)<br><br>P=0.0023	○	⊕	○	○	○	○	✡

9	Sterud[20] 2016 USA	Investigate the extent to which work-related factors contribute to the social gradient in LBP.	12,550 randomly drawn cohort from the general population in Norway aged 18 to 66 years	Group 1: university/college ≥4 years; group 2: university/college <4 years; group 3: upper secondary; group 4: incomplete upper secondary; group 5: elementary secondary.	Reported intensity of LBP during the 4 weeks before answering the questionnaire: cases were defined as respondents who reported being severely or somewhat afflicted at follow-up	3-year	All sample, OR: 0.609 95%CI: 0.524 to 0.708.<br><br>Sex based analysis for men and women	○	○	⊕	⊕	○	○	✡
10	Menezes Coasta[31] 2009 Australia	Describe the course of chronic LBP in an inception cohort and to identify prognostic markers at the onset of chronicity.	406 patients (aged 44.1±14.5 years) with recent onset chronic non-specific LBP. Participants were drawn from a larger cohort study of 973 consecutive patients with acute LBP (<2 weeks' duration) from primary care clinics in Sydney.	Categorize as no education beyond secondary school	Recovered if reported pain-free, and had no disability from LBP, and had returned to work for 30 consecutives days.	12 months	No education beyond secondary school crude OR= 0.65 (0.48 to 0.88) p=0.006, Adjusted OR=0.74 (0.54 to 1.00), p=0.05 Adjusted for previous sick leave due to LBP, risk of persistent pain, and pain related disability.<br><br>Recovery from LBP related disability, No education beyond secondary school, crude OR=0.72 (0.50 to 1.03), p=0.07	○	●	⊕	⊕	●	○	✸
11	Gurcay[22] 2009 Turkey	Assess the clinical course of patients with acute LBP throughout 12 weeks and to identify the prognostic factors for non-recovery in the short term.	91 patients with acute LBP (<3 weeks), a mean of 37.9+10.3 years.	Education as years	Patients were considered recovered if they rated pain intensity as 0 and scored <4 on the RMDQ.	1, 2, and 12 weeks.	Recovered group: 8.1±2.9 and non-recovered group: 8.1±3.6, p= 0.912	○	●	○	○	●	⊕	✸
12	Lehmann[32] 1993* USA	To develop a predictive model for occupational physician and the spine consultant for high-risk long term disability.	55 (37 men) patients aged between 18-65 (average 37.2+9.07) years presenting with acute LBP who have incurred work absences of more than 2 but less than 6 weeks duration.	Education in years. None of the participant had college degree.	Time until return to work	6 months	No correlation between disabling LBP/time until return to work and education (p=0.17).	⊕	●	○	○	●	⊕	✸
13	Gatchel[33] 1995 USA	Evaluate whether a comprehensive assessment of psychosocial measures is useful in characterizing those acute LBP patients	324 (ages: 35.2 ± 11.7 years) acute back patients of no more than 6 weeks.	No high school<br>High school<br>Some college or more	LBP patients who were back at work at 6 months versus those who were not because of the original back injury	6 months	These analyses revealed that the disabled group consisted of people tended to include those with lower EA, t(308) = 1.64, p < .10, relative to the nondisabled group.<br>Higher education group: 123	○	●	⊕	○	●	○	✸

		who subsequently develop chronic pain disability problems.					Lower education group: 185							
14	Gatchel[34] 1995 USA	Evaluate the predictive power of psychosocial and personal factors in identifying acute LBP to progress to chronic pain disability	421 patients (aged: 35.0 ± 11.5) seen at three clinics for an acute back pain episode of no more than 6 week	No high school and High school versus Some college or more	who were back at work at 12 months versus those who were not because of the original back injury	12 months	No significant difference between disabled group and non-disabled group with education.	○	●	○	○	●	○	✸
15	Silva[35] 2022 Brazil	Describe pain and disability trajectories in older adults with a new episode of LBP.	542 older adults, aged >55 years with a new episode of nonspecific LBP. Acute pain of <6 weeks and the individual has not sought care in health services owing to the LBP in the 6 months before the current.	Schooling level: low, medium and high	Pain intensity using NPRS and Disability (Roland-Morris Disability Questionnaire)	12 months	Low schooling level (proportion and 95%CI):<br>Pain trajectory: Pain recovery p= 0.03.<br><br>Disability trajectories:	○	○	○	○	⊕	○	✡
16	Seyedmehdi[36] 2015 Iran	Assess the power and quality of General Health Questionnaire (GHQ) for prediction of the odds of chronicity of acute LBP	511 workers (500 were male) complaining of a new LBP in the past two weeks from large rubber factory in 2011-2013 in the city of Yazd, Iran	Educational level: <Diploma and ≥ Diploma	Chronic low back pain status: group 1whose LBP lasted for <3 months and group 2 whose LBP lasted for ≥3	12 months	Unadjusted OR= 0.474 (0.294-0.758), p=0.002<br><br>Adjusted OR= 0.73 (0.42735 - 1.235)<br>Adjusted: age, sex, job parameters, smoking, BMI, exercise	○	○	○	⊕	○	○	✡
17	Turner[37] 2008 USA	To identify early predictors of chronic work disability after work-related back injury. Interviewed three weeks after submitting a lost worktime.	1885 (68% male) receiving work disability compensation from Washington State Department of Labor and Industries claims database July 2002 through April 2004,	-High school<br>-Less than high school<br>-Vocational or some college<br>-College	The primary outcome was wage replacement compensation for temporary total disability ("work disability") 12 months after claim submission.	12 months	Bivariate logistic regression analyses predicting 1-year work disability.<br><br>Having a college degree was crude OR=0.39 (0.21–0.75) but in the adjusted OR= 0.53 (0.23–1.18).	⊕	●	○	⊕	○	○	✸
18	Von Korff[38] 1991 USA	Improve understanding of the outcome of back pain in primary care patients. Three to six weeks after the index visit to the primary care clinic	1128 back pain patients with 3-6 weeks after an index visit of back pain recruited for back pain and follow up at 1 year.	College graduate<br>HS graduate<br><12 years	Severity of back pain (0 – 10 ratings) and total days of back pain during a 6-month interval	12 months	HS graduate versus college graduate: adj-OR= 1.85, p=0.005<br><12years versus college education: adj-OR= 3.17, p=0.004<br><br>Adjusted: age, gender, education, race, pain grade, pain	⊕	●	⊕	○	●	⊕	✸

		(via phone interview).					days, recency, disability payment							
19	Fliesser[39] 2018 Germany	Investigate associations between socioeconomic status indicators (education, job position, income, multidimensional index) and the genesis of chronic LBP.	352 people with intermittent pain aged between 18 and 65 years of age, from four medical clinics across Germany as part of a national study on low back pain. Participants already reporting chronic pain syndrome were excluded.	International Standard Classification of Education from 0 (less than primary education) to 5 (tertiary education).	Chronic Pain Grade Questionnaires: Pain disability, pain intensity, pain class	6-month	Pain disability Score Upper secondary education versus tertiary education B= 5.8, SE= 2.0, p <0.01** <br>Pain intensity score. Upper secondary education versus tertiary education B: 4.3, SE: 2.0 p=0.03* Adjusted for age and sex.	●	●	○	○	●	⊕	✸
20	Turner[40] 2013 USA	Examine whether its predictive ability of the Chronic Pain Risk Score could predict risk for chronic pain	521 adult patients initiating primary care for new episode of LBP from Group Health in Seattle. Patients who could not be reached within 14 days after this visit were excluded.	Highest grade of education completed.	Chronic pain risk model <br>4-month outcomes: chronic pain grade – algorithm incl. 3 pain severity and 3 pain interference.	4 months	Expanded Chronic Pain Risk Model adjusted OR (95% CI): 0.48 (0.28, 0.81), p<0.01 <br>Adjusting for all other variables: pain related outcomes, psychological opioid use	○	○	⊕	○	⊕	○	✵
**Subacute (>6 weeks)**														
21	Valencia[41] 2011 USA	Examined whether SES should be considered as mediator between the relationship of fear-avoidance beliefs and pain catastrophizing on disability, pain intensity and physical impairment	108 patients with acute or sub-acute LBP, which were recruited from university of Florida affiliated orthopedic physical therapy clinics.	- 1) less than high school, 2) graduated from high school, 3) some college, 4) graduated from college, 5) some postgraduate, and 6) completed post graduate degree.	Pain intensity using NPRS and disability with ODI.	1 and 6 months	SES did not predict pain intensity (Beta = -0.01, (std er: 0.01) P = 0.91) or disability (Beta = 0.75 (err: 0.62), P = 0.229) while controlling for pain catastrophizing <br>SES correlated with pain catastrophizing (r = -0.37, P < 0.01).	●	●	⊕	○	●	⊕	✸
22	Turner[42] 2006 USA	Examine whether worker demographics, pain, disability, and psychosocial variables assessed soon after work-related LBP disability onset, predict 6-month work disability.	1,068 workers, aged ≥18 years who submitted claims for work-related back pain and received at least 1 day of temporary total disability wage replacement (July 2002 to June 2003)	-Less than high school -High school -College or vocational/technical -College graduate	Pain intensity in the past week NPRS Physical disability was assessed by the RDQ.	6 months	Work disability Crude: - Less than high school 155 (15), OR: 0.99 (0.62–1.59) - High school 373 (35) OR: 1.00 <br>- College or vocational/ technical 454 (42), OR: 0.86 (0.61–1.22) - College graduate 86 (8), OR: 0.46 (0.22 – 0.97).	○	⊕	○	○	○	○	✵

23	Epping-Jordan[43]<br>1998<br>USA	Predictive relationship between pain intensity, disability and depressive symptoms	78 men, 31.9±7 years of age and 12.8±1.6 years of education with first onset low back pain 8±2 weeks, who were part of a research program studying progression to chronic LBP.	Years of education	Pain intensity using the Descriptor Differential Scale, and disability with Sickness Impact Profile,	2, 6, and 12 months	Correlation analysis: years of education were not significantly associated with pain intensity, disability, or depressive symptoms.	○	○	○	○	○	○	✡
24	Melloh[19]<br>2011<br>New Zealand	Identify factors that influence the progression of acute LBP or recurrent LBP after a pain-free period of at least 6 months to the persistent state at an early stage.	53 consecutive patients with acute LBP participated over the 6-month period	Higher and lower level of EA	Patients suffering from persistent LBP by functional limitation that is disabling at baseline or 3-week follow-up (10 ODI points) and still severely impacts after 6 weeks	6 months	OR= 0.491, 95%CI= 0.13 to 1.86	⊕	○	⊕	○	○	⊕	✡
25	Poiraudeau[23]<br>2006<br>France	Assess the outcome of subacute LBP to identify the characteristics of patients and physicians' beliefs about LBP.	266 Rheumatologists and 440 patients with subacute low back pain (of 4-12 weeks only).	No full-time education<br>Primary school<br>High school<br>Post-graduate	Persistence LBP: 'Has your low back pain persisted since your visit to your rheumatologist 3 months ago?'	3 months	OR= 0.917, 95%CI = 0.58 to 1.29, P=0.668	○	⊕	○	⊕	●	○	★
26	Williams[44]<br>1998<br>USA	Determine the extent to which job satisfaction predicts pain, psychological distress, and disability 6 months after an initial episode of LBP.	136 consecutive men, age between 18 and 50 years, that back pain (T6 or below) that had been present "on a daily previous 8±2 weeks.	Years of education	Pain (VAS) and disability (Sickness Impact Profile, Quality of Well-Being).	2 and 6 months	Zero-order correlations among six-month outcome measures were non-significant with years of education,	○	⊕	○	○	○	○	✡
27	Kongsted[45]<br>2015<br>Denmark	Identify LBP trajectories using LBP intensity and frequency measured once a week over 1 year and compare the results obtained using different analytical approaches.	1,082 participants consulting a health care provider for the first time due to their current episode of LBP and must not have attended another chiropractor within the last 3 months	No formal post-high school education, vocational training, higher education of <3 years, higher education of 3–4 years, higher education of >4 years,	Eight Subgroup to predict trajectory of back pain. From groups 1, Recovered, to group 8, moderate ongoing daily.	2 weeks, 3 months, and 12 months	Subgroups were not significantly associated with EA.<br>Subgroup 1 vs 5: OR=0.5, p=0.09	○	⊕	○	⊕	⊕	⊕	
28	Cats-Baril[46]<br><br>1991<br>USA	Develop a predictive risk model for low back pain disability better than	250 patients (aged,18-65 years) attending two secondary care for new episode of LBP,	Level of education	Whether a patient returns to work or becomes disabled, after 6 months	3 and 6 months	The discriminate function analysis identified EA (p<0.05) as predictive of disabled	○	●	●	⊕	⊕	○	★

		experienced specialist and generalist.	which has not prevented them from working for at least the last three months											
29	Karjalainen[47] 2003 Finland	Identify outcome determinants of subacute LBP.	164 employed patients with subacute (duration of pain 4–12 weeks) daily LBP recruited from 36 primary health care centers in the Helsinki metropolitan area	High school diploma	Sick leave because of back pain, intensity of pain (rated 0 to 10), back-specific perceived disability (ODI).	3, 6, and 12 months.	Univariate:<br>Intensity of pain = p<0.01<br>ODI = p=0.57<br>Multivariate: intensity of pain = p>0.05<br>Adjusted for all significant values in the first model: Age, gender, occupation, health status.	○	○	○	○	○	○	☆
	Key:	☆ Low	⊗ Moderate	★ High										

Figure 2: Percentage distribution of the types of bias in the quality appraisal criteria of the included studies.

Prognostic factors

Operational definitions and categories of EA were defined differently across studies. Thirteen studies[20,21,23,25,27,28,30,31,33,36,37,39,42] had EA according to a 12-year education grade (e.g., 12 years or less, 12 years or more). Three did not present adequate data for extracting years of education. Silva et al[35] categorized education as ‘low, medium, or high” with no specific details, and Valencia et al[41] combined income and education into a single index of socio-economic status. Turner et al [40] reported EA as highest grade of education completed without detailing the years.

Outcomes

Outcomes were broadly categorized into pain, disability or a combination of pain and disability. Pain was evaluated using 21 outcomes across 14 studies with the Numeric Pain Rating Scale (NPRS),[28,35,39,41,42] and Visual Analog Scale (VAS)[44] the most frequent. Disability was evaluated using 21 outcomes in 9 studies most commonly using the Oswestry Disability Index (ODI)[19,41,47] and Roland Morris Disability Questionnaire (RMDQ).[22,28,35] A combination of both pain and disability was evaluated in two studies.[22,31]

Meta-Analysis

Of the 29 articles from 27 studies, 9 articles[24,32,34,38,43–47] did not report adequate data (e.g., proportions, estimates) to permit statistical pooling and we were unsuccessful in contacting the original authors of those papers. Accordingly, data from 20 studies were available for meta-analysis.

EA as a predictor for the onset of new LBP

Figure 3 show the forest plot for pooled estimate for the association between EA and the onset of LBP. Three longitudinal inception cohort studies[25–27] (total N = 3,110) presented adequate data for pooling. The pooled effect of the three studies shows consistent evidence of no association between EA and new onset of LBP (OR = 0.93; 95%CI 0.75 to 1.15) with homogeneity ($I^2 < 0.1\%$).

Fig. 3. Forest plot of prognostic accuracy (odds ratio, OR) of educational attainment for predicting a) new onset, b) acute outcomes, and c) subacute outcomes in LBP.

EA as a prognostic variable for predicting outcome of acute LBP.

Eight studies (total N = 15,079) reporting pain as an outcome were pooled with low-moderate heterogeneity in effect sizes ($I^2 = 35\%$). Results supported a significant effect, in which higher EA predicts lower intensity LBP symptoms 3 months to 3 years after onset of acute LBP (OR = 0.54; 95%CI 0.44 to 0.67, Figure 3). Three of those studies reported adjusted estimates only, excluding those resulted in an equivocal shift in pooled effects using only the unadjusted estimates (OR = OR: 0.51, 95%CI: 0.37 to 0.70, I = 36%, Figure 4). Nine articles (8 cohorts, total N = 4,672 subjects) reported a pain-related disability outcome. Pooling similarly indicated that higher EA measured in the acute phase of LBP predicts lower pain-related disability 3 months to 12 months later (OR = 0.57; 95%CI 0.42 to 0.76, Figure 3) with moderate heterogeneity ($I^2 = 44\%$). Excluding the adjusted estimates from two of those studies again resulted in an equivocal shift in pooled effect (OR: 0.62, 95%CI: 0.51 to 0.77, Figure 4) but without heterogeneity ($I^2 = 0\%$). Of the three studies that could not be pooled, two studies [24,38] (moderate RoB, total N = 1,369) also reported significant associations between outcomes of acute LBP and EA. The third[32] (1 moderate RoB, N = 55 subjects) reported no association between EA and disabling LBP/time to return to work.

Fig. 4. Forest plot of the association of unadjusted back pain outcomes (acute and subacute) and educational attainment with pooled estimates and 95% confidence intervals.

EA as a prognostic variable for predicting outcome of subacute LBP.

Two (pain severity)[23,41] and three (pain-related disability)[19,41,42] studies predicting outcomes in subacute LBP could not be meaningfully pooled owing to high heterogeneity ($I^2 > 79\%$), inconsistent outcomes, and too few sources to permit moderator analysis. Accordingly, we proceeded with qualitative synthesis. For pain intensity as an outcome 4 of 6 studies (2 low[43,44] and 2 moderate[23,45] RoB, total N= 2,880) reported unadjusted (bivariate) estimates and indicated no association between EA and follow-up outcome. The remaining two studies (1 low[47] and 1 moderate[41] RoB, total N= 272) reported no significant association after adjusting for pain catastrophizing[41] or age, gender, occupation, health status.[47] For pain-related disability, 7 studies (1 low, 3 moderate and 2 high RoB studies, total N = 1,960) reported inconsistent evidence. Two studies[42,46] (total N = 1,504) found a significant negative association between EA and pain-related disability as measured with the RDQ[42] and ability to return to work[46]. Four other studies[42,44] (2 low,[19,47] 1 moderate[19] and 1 high[41] RoB, total N = 403) reported no association between EA and ODI[19,41,47] or sickness profile.[43]

Sex based analysis of educational attainment and low back pain outcomes.

Two studies analyzed data for potential differential effects of EA on LBP when disaggregated by sex. The two studies could not be pooled due to differences in case definitions. Zadro et al[26] studied new onset LBP and reported lower EA to be associated with increased proportion of new onset LBP in females only, with no significant effects in males. Sterud et al[20] evaluated outcomes in acute LBP and found no differential effect on outcome between sexes.

GRADE STATEMENT

Evidence profile of all included studies applied using GRADE is presented table 2.

EA and onset of LBP: On the basis of 3 studies, 1 moderate and 2 high RoB, with consistency in magnitude of effect, we find low-to-moderate confidence that EA has no association with onset of new LBP in adults when followed for at least 2 years.

EA and outcomes of acute LBP: On the basis of 9 of 11 studies, 6 moderate and 3 high RoB, we have moderate confidence that EA when collected at inception shows a significant association with LBP symptom severity measured at least 3 months later. We find low confidence based on 5 of 11 studies, 2 moderate and 3 high RoB of a similar association when the outcome is pain-related disability.

EA and outcomes of subacute LBP: On the basis of 7 of 9 studies, 2 low, 3 moderate, and 2 high RoB, we find inconsistent evidence and very low confidence on any association between EA and subsequent outcomes of pain severity when participants are incepted at the subacute (8-12 weeks from onset) stage. We find inconsistent evidence based on 3 of 5 studies (1 low, moderate and high RoB) and very low confidence for no association between EA and disability at least 3 months later. Significant heterogeneity in case definitions and effect sizes preclude more definitive findings.

Table 2: Evidence profile for the association between low back pain outcomes and educational attainment.

Low back pain and educational attainment findings		GRADE Variables					
**STUDIES**	**Association**	**Risk of Bias**	**Inconsistency**	**Indirectness**	**Imprecision**	**Publication bias**	**Certainty in the evidence***
Onset							
Zadro[26] Von Korff[25] George[27]	**x**	M=1 H=2	No Serious inconsistency	No Serious indirectness	No serious imprecision	Unlikely	⊕⊕⊕⊕ High certainty for evidence of no association between educational attainment and onset
Acute pain							
*Besen[24] *Karjalainen[47] , Starkweather,[30] Sterud[20], Silva,[35] Seyedmehdi[36], *Von Korff[38] , Fliesser[39] Turner,[40] Coasta[31]	**-**	H=3 M=6	Moderate inconsistencies	No Serious indirectness	No serious imprecision	Unlikely	⊕⊕⊕◯ Moderate certainty of association between low educational attainment and poor pain outcome in acute LBP
Coasta[31] , Seyedmehdi[36],	**x**	H=1 M=1					
Acute disability							
Moffett[29], Silva[35] Turner[37] Fliesser[39] Coasta[31]	**-**	M=2 H=3	Moderate inconsistencies	No Serious indirectness	No serious imprecision	Unlikely	⊕⊕◯◯ Low certainty of evidence for the association between low educational attainment and poor disability outcomes in acute LBP
Coasta[31], Gatchel[33,34] Grotle[621,28] , Gurcay[22] Turner[37], *Lehmann[32]	**x**	H=5 L=1					
Subacute pain							
Valencia[41], Karjalainen[47]	-	L=1 H=1	No Serious inconsistency	Moderate indirectness	No serious imprecision	Unlikely	⊕⊕⊕◯ Moderate certainty of evidence of no association between educational attainment and pain outcome in subacute LBP
Valencia[41], *Epping-Jordan,[43] Poiraudeau[23] *Williams[44] *Kongsted[45] , Karjalainen[47]	**x**	L=2 M=2 H=2					
Subacute disability							
Turner [42] *Cats-Baril[46]	**-**	M=1 H=1	Serious inconsistency	Moderate indirectness	No serious imprecision	Unlikely	⊕⊕◯◯ Very low certainty of evidence for the association between educational attainment and disability outcome in subacute LBP
Valencia[41], *Epping-Jordan[43] Karjalainen[47], Melloh,[19]	**x**	H=1 M=1 L=2					

Discussion

We have conducted a rigorous and systematic search, extraction, and pooling of effects to explore the associations between a key indicator of socioeconomic status (highest level of education completed) and each of onset of new LBP, outcomes of acute LBP, or outcomes of subacute LBP. This represents part of an ongoing set of reviews exploring the social determinants of health and their associations with LBP, conceptualized herein as evaluating whether lower EA (high school or less) functions as either a potential risk or prognostic factor for onset or outcome of LBP, respectively. As observational studies these are inherently

vulnerable to confounding bias from several other potential variables meaning causation should not be assumed. Based on the strength and effects of available evidence, we have moderate confidence in a significant negative *association* between EA and pain severity or disability outcomes of acute LBP in which higher EA may offer some protection against poor outcomes, low confidence that EA has no association with onset of new LBP, and very low confidence of any association between EA and outcome when starting from the subacute LBP stage.

While to our knowledge the synthesis of evidence related to new-onset LBP is novel, our results are largely consistent with those of other reviews in acute or subacute LBP, each of which included EA as part of a larger set of potential prognostic variables and few of which conducted meta-analyses. Previous LBP studies have failed to establish an association between EA and LBP outcomes for various reasons such as a small sample size to statistically power the study to detect effects,[42] lack of uniform study design, [48] and heterogeneous population, among others. For example, a review by Batista et al[48] reported that individuals with higher EA are less often affected by the occurrence of LBP. However, that review included multiple study designs that might have added noise to any estimates of effect. Cancelliere and colleagues[49] based on best evidence synthesis of systematic reviews on factors affecting return to work after injury or illness identified higher EA and socioeconomic status among factors associated with positive return-to-work outcomes. A similar review by Dionne and colleagues[50] included multiple study designs that could not allow establish prediction or causation, though that review concluded that people with lower EA are more likely to be affected by disabling LBP.

While it is tempting to ascribe mechanisms to either the finding of no association with onset or consistent association with outcome in LBP, any such attempt is necessarily speculative given the design of studies and the inability to feasibly conduct a randomized trial in which one arm remains uneducated. Accordingly, criteria to support cause-and-effect, most famously

described by Bradford-Hill[51,52] may never be fully realized. However, it also limits the impact of this work if no potential mechanisms are explored. EA is commonly included as part of the indices used to assign people to socioeconomic strata,[53,54] that also include variables such as annual household income and median neighborhood income. From a Bourdieusian perspective, each of these may be interpreted as inferring capital that can be converted to power across different social fields.[55] In the context of outcomes of LBP, possessing this type of social capital may permit easier access to effective care or alternative employment options for those of SES, meaning that research using outcomes such as work status may find those with economic or educational privilege have better outcomes. However, EA may be functioning more as a proxy for other influences on experiences of health and wellness outcomes. For example, higher EA may also signal higher health literacy, living in more affluent areas with easier access to schools, or family wealth. Lower EA may be associated with, amongst other things, experiences of school bullying, early parentification, poor mental health, or neighborhood poverty.[56] Each may also play a moderating or mediating role on health outcomes,[57] suggesting that these effects are very likely complex interactions between person- and society-level influences.

That EA showed no significant association with the onset of new LBP also demands further interpretation. Importantly, on the basis of only 3 studies of moderate-to-high RoB we cannot have more than low confidence in the finding, though the consistency from over 3,000 participants suggests a signal may be present. Intuitively we might expect that those with lower EA are also more likely to be in jobs that demand higher physical labour or more repetitive tasks that might increase the risk of musculoskeletal disorders like LBP. However, we can see prior evidence that appears to support the lack of association identified herein. For example, in a large population-level study of >74,000 U.S. adults aged 30-49, Zajacova and colleagues found a non-linear association between EA and pain, in which adults who started but did not

finish a post-secondary educational program reported higher prevalence of painful conditions than either those with completed post-secondary education *or* those with secondary education only.[58] There is also an abundance of evidence associating sedentariness or prolonged sitting, as may be more likely experienced by those with higher-level or managerial roles, as risk factors for low back pain. Further, amongst blue collar workers Lagersted-Olsen and colleagues found no association between daily time spent in a forward-bending posture and onset or aggravation of LBP over one year.[59] Accordingly, similar to our commentary on EA and LBP outcomes, any association between EA and LBP onset is likely similarly complex and it seems overly simplistic to suggest that lower education does or does not lead to LBP.

Limitations to the study include the inability to establish causation as previously described, though this is more a limitation of the overall field rather than this particular review. We were also unable to retrieve missing or under-reported data by contacting authors, meaning that some potentially relevant data have not been included in the meta-analyses that may otherwise change the results. All of the included studies were written in English or had a professional English translation available, meaning we may have missed data from work published in non-English journals. Finally, despite our best efforts not all studies reported EA in a way that permitted easy dichotomization into the 12-year categories. We made our best estimates when based on reporting in the manuscript when grouping results into one of these two categories, though acknowledge that some errors may have been made.

REFERENCE

1. Vos T, Abajobir A, Abate K, Abbafati C, Abbas K et al. Global, regional, and national incidence, prevalence, and years lived with disability for 328 diseases and injuries for 195 countries, 1990-2016: a systematic analysis for the Global Burden of Disease Study 2016. *Lancet*. 2017;390(10100):1211-1259. doi:10.1016/S0140-6736(17)32154-2
2. Cohen SP, Vase L, Hooten WM. Chronic pain: an update on burden, best practices, and new advances. *Lancet*. 2021;397(10289):2082-2097. doi:10.1016/S0140-6736(21)00393-7
3. Angarita-Fonseca A, Trask C, Shah T, Bath B. Stable prevalence of chronic back disorders across gender, age, residence, and physical activity in Canadian adults from 2007 to 2014. *BMC Public Health*. 2019;19(1):1121. doi:10.1186/s12889-019-7395-8
4. Croft PR, Macfarlane GJ, Papageorgiou AC, Thomas E, Silman AJ. Outcome of low back pain in general practice: a prospective study. *BMJ*. 1998;316(7141):1356-1359. doi:10.1136/bmj.316.7141.1356
5. Hestbaek L, Leboeuf-Yde C, Manniche C. Low back pain: what is the long-term course? A review of studies of general patient populations. *Eur Spine J*. 2003;12(2):149-165. doi:10.1007/s00586-002-0508-5
6. Crook J, Milner R, Schultz IZ, Stringer B. Determinants of occupational disability following a low back injury: a critical review of the literature. *J Occup Rehabil*. 2002;12(4):277-295. doi:10.1023/a:1020278708861
7. Schultz IZ, Crook J, Meloche GR, et al. Psychosocial factors predictive of occupational low back disability: towards development of a return-to-work model. *Pain*. 2004;107(1-2):77-85. doi:10.1016/j.pain.2003.09.019
8. Turk DC. A diathesis-stress model of chronic pain and disability following traumatic injury. *Pain Res Manag*. 2002;7(1):9-19. doi:10.1155/2002/252904
9. Ramond A, Bouton C, Richard I, et al. Psychosocial risk factors for chronic low back pain in primary care-a systematic review. *Fam Pract*. 2011;28(1):12-21. doi:10.1093/fampra/cmq072
10. Glass TA, Bilal U. Are neighborhoods causal? Complications arising from the ‘stickiness’ of ZNA. *Soc Sci Med*. 2016;166:244-253. doi:10.1016/j.socscimed.2016.01.001
11. Carter MA, Dubois L, Tremblay MS, Taljaard M, Jones BL. Trajectories of Childhood Weight Gain: The Relative Importance of Local Environment versus Individual Social and Early Life Factors. *PLoS One*. 2012;7(10). doi:10.1371/journal.pone.0047065
12. Ramage-Morin PL, Gilmour H. Chronic pain at ages 12 to 44. *Health Rep*. 2010;21(4):53-61.
13. Walton DM, Macdermid JC, Giorgianni AA, Mascarenhas JC, West SC, Zammit CA. Risk factors for persistent problems following acute whiplash injury: update of a systematic review and meta-analysis. *J Orthop Sports Phys Ther*. 2013;43(2):31-43. doi:10.2519/jospt.2013.4507
14. Steenstra IA, Verbeek JH, Heymans MW, Bongers PM. Prognostic factors for duration of sick leave in patients sick listed with acute low back pain: A systematic review of the literature. *Occup Environ Med*. 2005;62(12):851-860. doi:10.1136/oem.2004.015842
15. Agnello A, Brown T, Desroches S, Welling U, Walton D. Can we identify people at risk of non-recovery after acute occupational low back pain? Results of a review and

higher-order analysis. *Physiotherapy Canada*. 2010;62(1):9-16. doi:10.3138/physio.62.1.9
16. Janwantanakul P, Sitthipornvorakul E, Paksaichol A. Risk factors for the onset of nonspecific low back pain in office workers: A systematic review of prospective cohort studies. *J Manipulative Physiol Ther*. 2012;35(7):568-577. doi:10.1016/j.jmpt.2012.07.008
17. Terwee CB, Mokkink LB, Knol DL, Ostelo RWJG, Bouter LM, de Vet HCW. Rating the methodological quality in systematic reviews of studies on measurement properties: a scoring system for the COSMIN checklist. *Qual Life Res*. 2012;21(4):651-657. doi:10.1007/s11136-011-9960-1
18. Deeks J, Higgins J, Altman D. Analyzing data and undertaking meta-analyses. In: *Higgins J, Green S, Editors. Cochrane Handbook for Systematic Reviews of Intervention Version 5.0.0.* Wiley; 2008.
19. Melloh M, Elfering A, Presland CE, et al. Predicting the transition from acute to persistent low back pain. *Occup Med (Chic Ill)*. 2011;61(2):127-131. doi:10.1093/occmed/kqq194
20. Sterud T, Johannessen HA, Tynes T. Do work-related mechanical and psychosocial factors contribute to the social gradient in low back pain? A 3-year follow-up study of the general working population in Norway. *Spine (Phila Pa 1976)*. 2016;41(13):1089-1095. doi:10.1097/BRS.0000000000001451
21. Grotle M, Brox JI, Veierød MB, Glomsrød B, Lønn JH, Vøllestad NK. Clinical course and prognostic factors in acute low back pain: Patients consulting primary care for the first time. *Spine (Phila Pa 1976)*. 2005;30(8):976-982. doi:10.1097/01.brs.0000158972.34102.6f
22. Gurcay E, Bal A, Eksioglu E, Esen Hasturk A, Gurhan Gurcay A, Cakci A. Acute low back pain: Clinical course and prognostic factors. *Disabil Rehabil*. 2009;31(10):840-845. doi:10.1080/09638280802355163
23. Poiraudeau S, Rannou F, Le Henanff A, et al. Outcome of subacute low back pain: Influence of patients' and rheumatologists' characteristics. *Rheumatology*. 2006;45(6):718-723. doi:10.1093/rheumatology/kei231
24. Besen E, Young AE, Shaw WS. Returning to Work Following Low Back Pain: Towards a Model of Individual Psychosocial Factors. *J Occup Rehabil*. 2015;25(1):25-37. doi:10.1007/s10926-014-9522-9
25. Von Korff M, Resche L Le, Dworkin SF. First onset of common pain symptoms: a prospective study of depression as a risk factor. *Pain*. 1993;55(2):251-258. doi:10.1016/0304-3959(93)90154-H
26. Zadro JR, Shirley D, Pinheiro MB, et al. Does educational attainment increase the risk of low back pain when genetics are considered? A population-based study of Spanish twins. *Spine Journal*. 2017;17(4):518-530. doi:10.1016/j.spinee.2016.10.021
27. George SZ, Childs JD, Teyhen DS, et al. Predictors of occurrence and severity of first time low back pain episodes: Findings from a military inception cohort. *PLoS One*. 2012;7(2). doi:10.1371/journal.pone.0030597
28. Grotle M, Brox JI, Glomsrød B, Lønn JH, Vøllestad NK. Prognostic factors in first-time care seekers due to acute low back pain. *European Journal of Pain*. 2007;11(3):290-298. doi:10.1016/j.ejpain.2006.03.004
29. Klaber Moffett JA, Underwood MR, Gardiner ED. Socioeconomic status predicts functional disability in patients participating in a back pain trial. *Disabil Rehabil*. 2009;31(10):783-790. doi:10.1080/09638280802309327

30. Starkweather AR, Lyon DE, Kinser P, et al. Comparison of Low Back Pain Recovery and Persistence: A Descriptive Study of Characteristics at Pain Onset. *Biol Res Nurs*. 2016;18(4):401-410. doi:10.1177/1099800416631819
31. Menezes Costa LDC, Maher CG, McAuley JH, et al. Prognosis for patients with chronic low back pain: Inception cohort study. *BMJ (Online)*. 2009;339(7725):850. doi:10.1136/bmj.b3829
32. Lehmann TR, Spratt KF, Lehmann KK. Predicting long-term disability in low back injured workers presenting to a spine consultant. *Spine (Phila Pa 1976)*. 1993;18(8):1103-1112. doi:10.1097/00007632-199306150-00023
33. Gatchel RJ, Polatin PB, Kinney RK. Predicting Outcome of Chronic Back Pain Using Clinical Predictors of Psychopathology: A Prospective Analysis. *Health Psychology*. 1995;14(5):415-420. doi:10.1037/0278-6133.14.5.415
34. Gatchel RJ, Polatin PB, Mayer TG. The dominant role of psychosocial risk factors in the development of chronic low back pain disability. *Spine (Phila Pa 1976)*. 1995;20(24):2702-2709.
35. Silva JP da, de Jesus-Moraleida FR, Felício DC, et al. Trajectories of pain and disability in older adults with acute low back pain: Longitudinal data of the BACE-Brazil cohort. *Braz J Phys Ther*. 2022;26(1). doi:10.1016/j.bjpt.2021.100386
36. Seyedmehdi SM, Dehghan F, Ghaffari M, et al. Effect of general health status on chronicity of low back pain in industrial workers. *Acta Med Iran*. 2016;54(3):211-217.
37. Turner JA, Franklin G, Fulton-Kehoe D, et al. ISSLS prize winner: early predictors of chronic work disability: a prospective, population-based study of workers with back injuries. *Spine (Phila Pa 1976)*. 2008;33(25):2809-2818.
38. Von Korff M, Deyo RA, Cherkin D, Barlow W. Back pain in primary care: Outcomes at 1 year. *Spine (Phila Pa 1976)*. 1991;18(7):855-862. doi:10.1097/00007632-199306000-00008
39. Fliesser M, De Witt Huberts J, Wippert PM. Education, job position, income or multidimensional indices? Associations between different socioeconomic status indicators and chronic low back pain in a German sample: A longitudinal field study. *BMJ Open*. 2018;8(4):1-7. doi:10.1136/bmjopen-2017-020207
40. Turner JA, Shortreed SM, Saunders KW, Leresche L, Berlin JA, Korff M Von. Optimizing prediction of back pain outcomes. *Pain*. 2013;154(8):1391-1401. doi:10.1016/j.pain.2013.04.029
41. Valencia C, Robinson ME, George SZ. Socioeconomic Status Influences the Relationship between Fear-Avoidance Beliefs Work and Disability. *Pain Medicine*. 2011;12(2):328-336. doi:10.1111/j.1526-4637.2010.01024.x
42. Turner JA, Franklin G, Fulton-Kehoe D, et al. Worker recovery expectations and fear-avoidance predict work disability in a population-based workers' compensation back pain sample. *Spine (Phila Pa 1976)*. 2006;31(6):682-689. doi:10.1097/01.brs.0000202762.88787.af
43. Epping-Jordan JAE, Williams RA, Pruitt SD, et al. Transition to chronic pain in men with low back pain: Predictive relationships among pain intensity, disability, and depressive symptoms. *Health Psychology*. 1998;17(5):421-427. doi:10.1037/0278-6133.17.5.421
44. Williams RA, Pruitt SD, Doctor JN, et al. The contribution of job satisfaction to the transition from acute to chronic low back pain. *Arch Phys Med Rehabil*. 1998;79(4):366-374. doi:10.1016/S0003-9993(98)90135-6
45. Kongsted A, Kent P, Hestbaek L, Vach W. Patients with low back pain had distinct clinical course patterns that were typically neither complete recovery nor constant

pain. A latent class analysis of longitudinal data. *Spine Journal*. 2015;15(5):885-894. doi:10.1016/j.spinee.2015.02.012

46. Cats-Baril WL, Frymoyer JW. Identifying Patients at Risk of Becoming Disabled Because of Low-Back Pain_The Vermont Rehabilitation Engineering Center Predict. *Spine (Phila Pa 1976)*. 1991;16(6):605-607.
47. Karjalainen K, Malmivaara A, Mutanen P, Pohjolainen T, Roine R, Hurri H. Outcome Determinants of Subacute Low Back Pain. *Spine (Phila Pa 1976)*. 2003;28(23):2634-2640. doi:10.1097/01.BRS.0000099097.61495.2E
48. Batista AA de S, Henschke N, Oliveira VC. Prevalence of low back pain in different educational levels: a systematic review. *Fisioterapia em Movimento*. 2017;30(suppl 1):351-361. doi:10.1590/1980-5918.030.s01.ar04
49. Cancelliere C, Donovan J, Stochkendahl MJ, et al. Factors affecting return to work after injury or illness: Best evidence synthesis of systematic reviews. *Chiropr Man Therap*. 2016;24(1). doi:10.1186/s12998-016-0113-z
50. Dionne CE, Von Korff M, Koepsell TD, Deyo RA, Barlow WE, Checkoway H. Formal education and back pain: A review. *J Epidemiol Community Health (1978)*. 2001;55(7):455-468. doi:10.1136/jech.55.7.455
51. Fedak KM, Bernal A, Capshaw ZA, Gross S. Applying the Bradford Hill criteria in the 21st century: how data integration has changed causal inference in molecular epidemiology. *Emerg Themes Epidemiol*. 2015;12:14. doi:10.1186/s12982-015-0037-4
52. HILL AB. THE ENVIRONMENT AND DISEASE: ASSOCIATION OR CAUSATION? *Proc R Soc Med*. 1965;58(5):295-300.
53. Lynch J, Kaplan G. Socioeconomic position. In: Edition 1st, ed. *Berkman LF, Kawachi I, Eds. Social Epidemiology*. Oxford University Press; 2000:13–35.
54. Liberatos P, Link BG, Kelsey JL. The measurement of social class in epidemiology. *Epidemiol Rev*. 1988;10:87-121. doi:10.1093/oxfordjournals.epirev.a036030
55. Bourdieu P. *La Reproduction*. Less Editi.; 1970.
56. Nieuwenhuis J, Kleinepier T, van Ham M. The Role of Exposure to Neighborhood and School Poverty in Understanding Educational Attainment. *J Youth Adolesc*. 2021;50(5):872-892. doi:10.1007/s10964-021-01427-x
57. Zajacova A, Lawrence EM. The Relationship Between Education and Health: Reducing Disparities Through a Contextual Approach. *Annu Rev Public Health*. 2018;39:273-289. doi:10.1146/annurev-publhealth-031816-044628
58. Zajacova A, Rogers RG, Grodsky E, Grol-Prokopczyk H. The Relationship Between Education and Pain Among Adults Aged 30-49 in the United States. *J Pain*. 2020;21(11-12):1270-1280. doi:10.1016/j.jpain.2020.03.005
59. Lagersted-Olsen J, Thomsen BL, Holtermann A, Søgaard K, Jørgensen MB. Does objectively measured daily duration of forward bending predict development and aggravation of low-back pain? A prospective study. *Scand J Work Environ Health*. 2016;42(6):528-537. doi:10.5271/sjweh.3591

 Click here to access/download;Figure;Fig 1.tif

Fig. 1: Flowchart of study selection and inclusion.

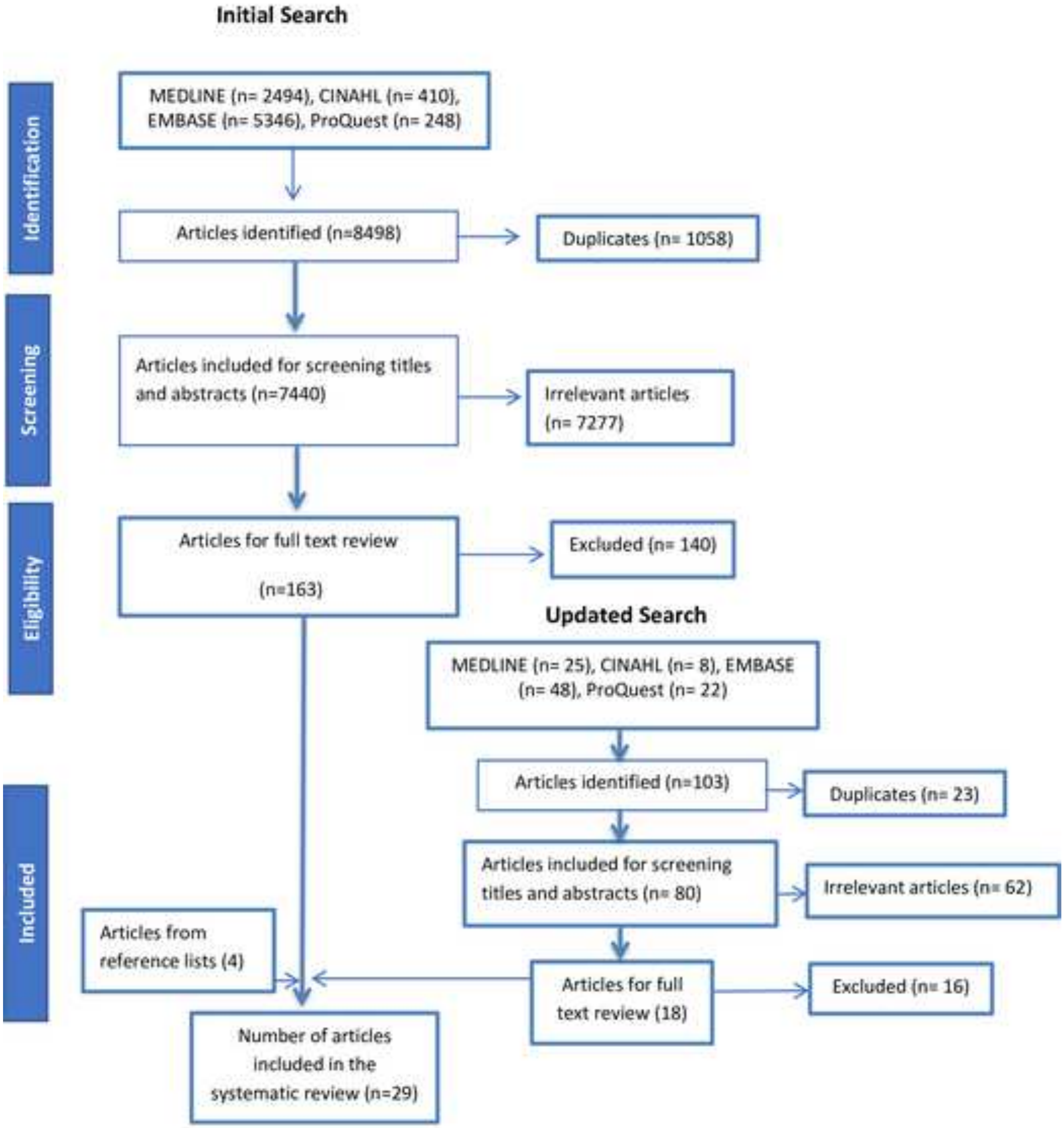

Figure 2

Click here to access/download;Figure;Fig 2.tif

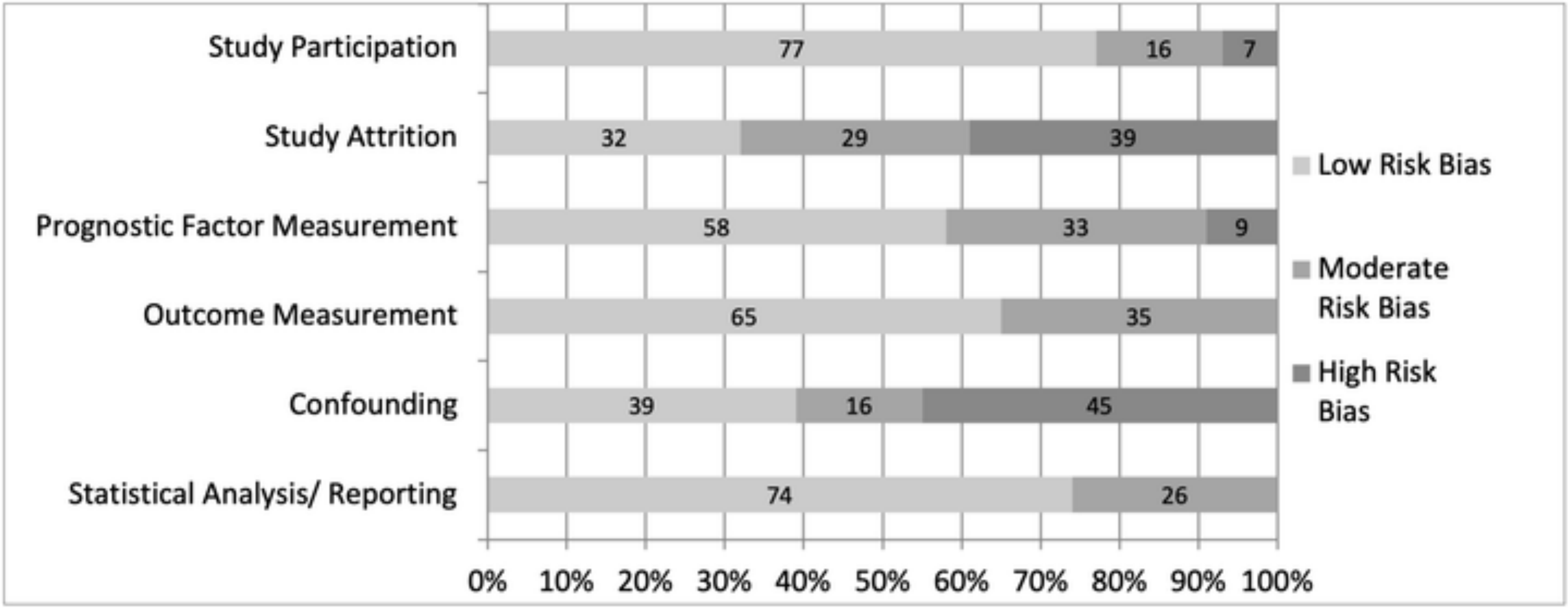

Figure 3

Click here to access/download;Figure;Fig 3.tif

Study	Back Pain	Education	Odds ratio [95% CI]	p-Value	% weight
**A. New onset**					
Zadro 2017	Onset back pain	12 years	0.80 [0.400, 1.60]	0.528	9.69
Von Korf 1993	Onset back pain	12 years	0.976 [0.403, 2.364]	0.957	5.95
George 2012	Onset back pain	12 years	0.940 [0.743, 1.188]	0.603	84.36
**Random Effects Model; z= -0.687; Test for Heterogeneity: I^2= 0%, p<0.905**			0.927 [0.747, 1.150]	0.492	
**B. Acute outcomes**	**Pain**				
Menendez-Coasta 2009	Pain free and had return to work	12 years	0.650 [0.480, 0.88]	0.005	23.88
Fliesser 2018	Chronic Pain Grade Q: Pain score	12 years	0.014 [0.00, 0.686]	0.032	0.31
Gurcay 2009	Recovered: VAS=0 & RMQ=<4	12 years	0.723 [0.194, 2.696]	1.00	2.64
Seyedmehdi 2015	Chronic: $\geq$3 month	12 years	0.474 [0.295, 0.761]	0.002	14.46
Silva 2022	NPRS	Low, medium & high	0.424 [0.203, 0.885]	0.022	7.44
Starkweather 2016	pain resolved in 6 weeks onset	12 years	0.147 [0.041, 0.532]	0.004	2.74
Sterud 2026	intensity of LBP <4 weeks	12 years	0.609 [0.524, 0.708]	0.001	36.19
Turner 2013	Chronic Pain Risk: severity & Interference	Highest	0.480 [0.282, 0.816]	0.007	12.34
**Random Effects Model; z= -5.507; Test for Heterogeneity: I^2= 35%, p= 0.146**			0.538 [0.432, 0.671]	0.001	
	**Disability**				
Menendez-Coasta 2009	Pain free and had return to work	12 years	0.720 [0.502, 1.033]	0.075	19.78
Fliesser 2018	Chronic Pain Grade Q: Disability score	12 years	0.003 [0.000, 0.151]	0.004	0.53
Gatchel 1995	Disabled did not return to work	12 years	0.707 [0.467, 1.071]	0.102	18.01
Grotle 2007	Recovered: $\leq$4 RMQ	12 years	0.650 [0.239, 1.765]	0.398	6.44
Gurcay 2009	Recovered: VAS=0 & RMQ=<4	12 years	0.723 [0.194, 2.696]	0.630	4.10
Moffet 2009	RMQ	20 years	0.364 [0.222, 0.597]	0.001	15.53
Silva 2022	RMQ	Low, medium & high	0.519 [0.316, 0.852]	0.01	15.50
Turner 2008	Disability: wage replacement	12 years	0.390 [0.206, 0.737]	0.004	11.95
**Random Effects Model; z= -3.782; Test for Heterogeneity: I^2= 44%, p= 0.038**			0.565 [0.420, 0.759]	0.001	
**C. Subacute outcomes**	**Pain**				
Poiraudeau 2006	Persistent pain: 3 months	12 years	0.917 [0.604, 1.390]	0.682	40.71
Valencia 2011	NPRS	Education with Income	0.236 [0.112, 0.498]	0.001	35.01
**Random Effects Model; z= -1.427; Test for Heterogeneity: I^2= 79%, p= 0.081**			0.490 [0.184, 1.306]	0.154	
	**Disability**				
Melloh 2011	Persistent: 10 ODI points 6 weeks	High & low	0.491 [0.130, 1.860]	0.295	24.28
Turner 2006	RDQ	12 years	0.460 [0.219, 0.966]	0.04	55.17
Valencia 2011	ODI	Education with Income	2.117 [0.628, 7.136]	0.226	44.83
**Random Effects Model; z=-0.122; Test for Heterogeneity: I^2= 77%, p= 0.036**			0.912 [0.206, 4.038]	0.903	
**Overall: Random Effects Model; z= -6.728; Test for Heterogeneity: I^2=80%, p=0.036**			0.685 [0.613, 0.765]	0.001	

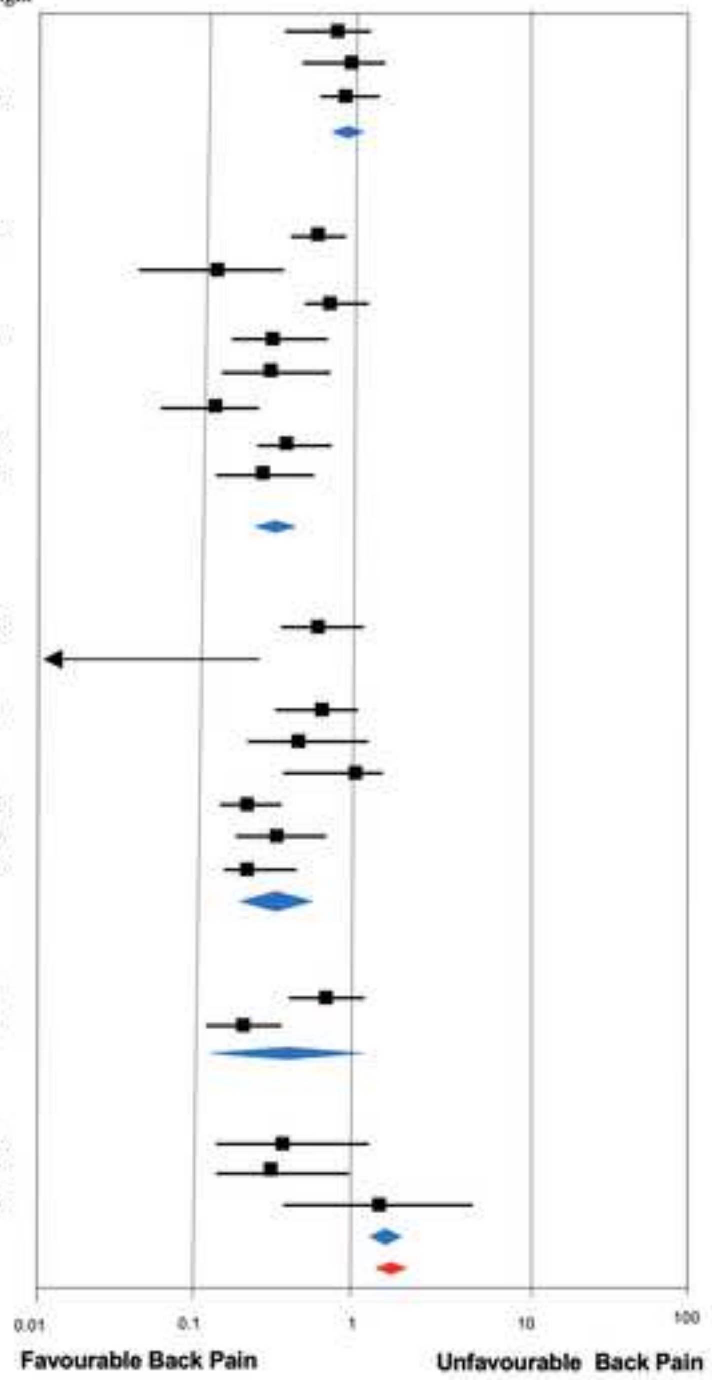

Figure 4

Click here to access/download;Figure;Fig 4.tif

Study	Back Pain	Education	Statistics: Odds ratio [95% CI]	p-Value	% weight
**A. Acute outcomes**					
	**Unadjusted Pain**				
Seyedmehdi 2015	Chronic: ≥3 month	12 years	0.474 [0.295, 0.761]	0.002	24.85
Silva 2022	NPRS	Low, medium & high	0.424 [0.203, 0.885]	0.022	13.78
Starkweather 2016	Pain resolved in 6 weeks onset	12 years	0.147 [0.041, 0.532]	0.004	5.36
Sterud 2026	Intensity of LBP < 4 weeks	12 years	0.609 [0.524, 0.708]	0.001	50.86
Gurcay	Recovered: VAS=0 & RMQ=<4	12 years	0.723 [0.194, 2.696]	0.630	5.15
**Random Effects Model; z= -4.228; Test for Heterogeneity: I^2= 36%, p= 0.179**			0.509 [0.372, 0.696]	0.001	
	**Unadjusted Disability**				
Menendez-Coasta 2009	Pain-free and had return to work	12 years	0.720 [0.502, 1.033]	0.075	33.36
Gatchet 1995	Disabled, did not return to work	12 years	0.707 [0.467, 1.071]	0.102	25.35
Grotle 2007	Recovered: ≤4 RMQ	12 years	0.650 [0.239, 1.765]	0.398	4.36
Silva 2022	RMQ	Low, medium & high	0.519 [0.316, 0.852]	0.010	17.68
Turner 2008	Disability: wage replacement	12 years	0.390 [0.206, 0.737]	0.004	10.75
Gurcay 2009	Recovered: VAS=0 & RMQ=<4	12 years	0.723 [0.194, 2.696]	0.630	2.52
**Random Effects Model; z= -4.422; Test for Heterogeneity: I^2= 0%, p= 0.698**			0.623 [0.506, 0.768]	0.001	

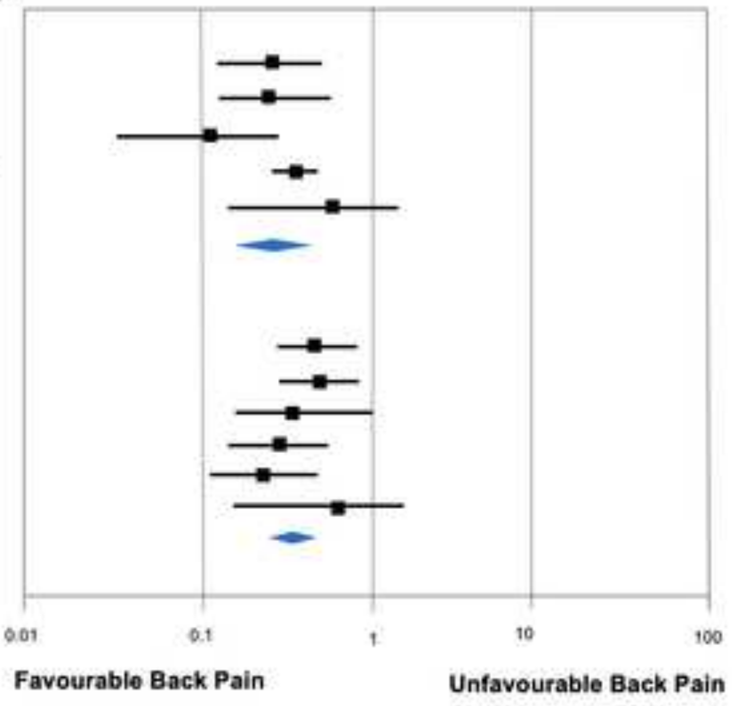

Appendix A: search strategy

Click here to access/download
Supporting Information
Appendix A Search Strategy.docx

Click here to access/download
Supporting Information
PRISMA_2020_checklist.docx
