# Supplementary material for: Is educational attainment associated with the onset and outcomes of low back pain? a systematic review and meta-analysis
Source: PLoS One. 2024 Aug 13;19(8):e0308625. doi: 10.1371/journal.pone.0308625 (PMC11321572; doi:10.1371/journal.pone.0308625)
Supplement: Attachment — Submitted filename: Letter with Response to Comments.docx [file pone.0308625.s004.docx]

June 13^th^, 2024

The Academic Editor,

PLOS ONE

**Response to Editors & Reviewer Comments**: Is educational attainment associated with the onset and outcomes of low back pain? A systematic review and meta-analysis (PONE-D-24-08660).

The authors sincerely thank the Academic Editor for finding our work worthy of consideration in PLOS ONE and for the opportunity to revise our submission. Also, we appreciate the reviewers for their thoughtful comments and suggestions to improve the manuscript. We have addressed the comments raised as follows:

Journal requirements:

When submitting your revision, we need you to address these additional requirements.

Comment: 1. Please ensure that your manuscript meets PLOS ONE's style requirements, including those for file naming. The PLOS ONE style templates can be found at

<https://journals.plos.org/plosone/s/file?id=wjVg/PLOSOne_formatting_sample_main_body.pdf> and

<https://journals.plos.org/plosone/s/file?id=ba62/PLOSOne_formatting_sample_title_authors_affiliations.pdf>

**Response**: The manuscript and all accompanying files are in accordance with PLOS ONE’s style requirements, and all named accordingly.

Comment: 2. We note that your Data Availability Statement is currently as follows: [The data underlying the results presented in the study are available within the manuscript.]

Please confirm at this time whether or not your submission contains all raw data required to replicate the results of your study. Authors must share the “minimal data set” for their submission. PLOS defines the minimal data set to consist of the data required to replicate all study findings reported in the article, as well as related metadata and methods (https://journals.plos.org/plosone/s/data-availability#loc-minimal-data-set-definition).

For example, authors should submit the following data:

- The values behind the means, standard deviations and other measures reported;

- The values used to build graphs;

- The points extracted from images for analysis.

Authors do not need to submit their entire data set if only a portion of the data was used in the reported study.

If your submission does not contain these data, please either upload them as Supporting Information files or deposit them to a stable, public repository and provide us with the relevant URLs, DOIs, or accession numbers. For a list of recommended repositories, please see <https://journals.plos.org/plosone/s/recommended-repositories>.

If there are ethical or legal restrictions on sharing a de-identified data set, please explain them in detail (e.g., data contain potentially sensitive information, data are owned by a third-party organization, etc.) and who has imposed them (e.g., an ethics committee). Please also provide contact information for a data access committee, ethics committee, or other institutional body to which data requests may be sent. If data are owned by a third party, please indicate how others may request data access.

**Response**: We are confirming that the submission contains all raw data required to replicate the results of your study, as stated on the title page line 20 to 21 “Data Availability Statement: The raw data involved in this analysis and required to replicate the results are available within the manuscript.”

Comment: 3. Please include captions for your Supporting Information files at the end of your manuscript, and update any in-text citations to match accordingly. Please see our Supporting Information guidelines for more information: <http://journals.plos.org/plosone/s/supporting-information>.

**Response**: There are no supporting documents needed to be added to the submission.


Reviewers' comments:

Reviewer's Responses to Questions

**Comments to the Author**

1. Is the manuscript technically sound, and do the data support the conclusions?

The manuscript must describe a technically sound piece of scientific research with data that supports the conclusions. Experiments must have been conducted rigorously, with appropriate controls, replication, and sample sizes. The conclusions must be drawn appropriately based on the data presented.

Reviewer #1: Yes

Reviewer #2: Yes

**Response**: Thank you for agreeing with the technical quality of our manuscript, including that the data support the conclusion made.

2. Has the statistical analysis been performed appropriately and rigorously?

Reviewer #1: N/A

Reviewer #2: Yes

**Response**: As agreed, the data undertake a meta-analysis using random effect to explore the predictive value of educational attainment and low back pain. Sensitivity analysis was undertaken to explore the effect of moderating variables as recommended. While variables with high heterogeneity were analyzed qualitatively. A summary of evidence was presented using GRADE.

3. Have the authors made all data underlying the findings in their manuscript fully available?

The [PLOS Data policy](http://www.plosone.org/static/policies.action#sharing) requires authors to make all data underlying the findings described in their manuscript fully available without restriction, with rare exception (please refer to the Data Availability Statement in the manuscript PDF file). The data should be provided as part of the manuscript or its supporting information, or deposited to a public repository. For example, in addition to summary statistics, the data points behind means, medians and variance measures should be available. If there are restrictions on publicly sharing data—e.g. participant privacy or use of data from a third party—those must be specified.

Reviewer #1: Yes

Reviewer #2: Yes

**Response**: We have confirmed as well that all data needed to replicate the findings are here within the manuscript.

4. Is the manuscript presented in an intelligible fashion and written in standard English?

PLOS ONE does not copyedit accepted manuscripts, so the language in submitted articles must be clear, correct, and unambiguous. Any typographical or grammatical errors should be corrected at revision, so please note any specific errors here.

Reviewer #1: Yes

Reviewer #2: Yes

**Response**: Despite the unanimous agreement between the reviewers on the intelligible fashion and standard language used to write the manuscript. We have thoroughly revised the manuscript for any typographical or grammatical errors and have made minor revisions to improve readability throughout.

5. Review Comments to the Author

Please use the space provided to explain your answers to the questions above. You may also include additional comments for the author, including concerns about dual publication, research ethics, or publication ethics. (Please upload your review as an attachment if it exceeds 20,000 characters)

Reviewer #1: This is a systematic review with meta-analysis on a very relevant topic, both due to the high worldwide prevalence of the outcome (LBP) and the significant importance of the exposure (educational attainment). Therefore, acquiring knowledge about the association between educational attainment and LBP is extremely necessary. However, despite generally following the recommendations of PRISMA 2020 (whose updated reference was not provided), some items related to the quality of SR advocated by the AMSTAR 2 tool were not met. For example, I missed the inclusion of articles in languages other than English, or at least a plausible justification for the exclusion of other languages. It was also not stated whether experts in the field were sought for suggestions on the inclusion of other studies. The PRISMA flowchart presented is not the currently recommended. The recommendation of an important item from AMSTAR 2, item 7 (Did the review authors provide a list of excluded studies and justify the exclusions?), was not fulfilled. The same applies to items 15 (If they performed quantitative synthesis did the review authors carry out an adequate investigation of publication bias (small study bias) and discuss its likely impact on the results of the review?) and 16 (Did the review authors report any potential sources of conflict of interest, including any funding they received for conducting the review?) of AMSTAR 2. Finally, regarding the discussion, I felt that the explanation of the strong points of this systematic review with meta-analysis and future proposals for development was missing.

**Response**:

1. The updated reference for PRISMA 2020 is now included in the citation.
2. We have included all language of publication as stated in the selection criteria (line: 148). While our search did not return any hits that appeared relevant but were written in another language, we also acknowledge that the databases we searched tend to favour English-language journals. This work is part of a larger body of research led by leading academics in the field of spinal (low back and neck) pain who are well-versed in the field and are unaware of other citations missed here, though admittedly that offers only a modicum of confidence in the exhaustiveness of our yield. The search strategy was extensively developed and applied with the help of a Health Science librarian to maximize the likelihood that all relevant studies are captured. While is seems unlikely that important work has been missed, we have nonetheless alluded to this potential limitation with the following addition to the Limitations section: “We did not include studies published in a language other than English or without a formal English translation available, raising the risk that we have missed data from work published in non-English journals that may influence our results.”.
3. The PRISMA flowchart is now updated according to the recommended PRISMA 2020 guideline (Page et al 2021).
4. The recommendation of an important item from AMSTAR 2,
   a. Item 7 (Did the review authors provide a list of excluded studies and justify the exclusions?), was not fulfilled.

*Response:* The updated PRISMA flowchart included the list of reasons and the number of studies excluded (see Fig 1Flow diagram.tif).

b. Item 15 (If they performed quantitative synthesis did the review authors carry out an adequate investigation of publication bias (small study bias) and discuss its likely impact on the results of the review?) and

*Response:* Acknowledging that assessing publication is a difficult task in meta-analysis with few studies. According to Cochrane’s Recommendations on testing for funnel plot asymmetry: “As a rule of thumb, tests for funnel plot asymmetry should be used only when there are at least 10 studies included in the meta-analysis, because when there are fewer studies the power of the tests is too low to distinguish chance from real asymmetry” (<https://handbook-5-1.cochrane.org/chapter_10/10_4_3_1_recommendations_on_testing_for_funnel_plot_asymmetry.htm>).

This considering that we did not have any meta-analysis that included at least 10 studies, we are unable to perform a funnel plot to test for publication bias and this is noted in as one of the study’s limitations on page 24 line 449 to 450. “Additionally, due the limited number of studies (fewer than 10) included in the meta-analysis, analysis of publication bias may be inappropriate.(Lau et al., 2006)“

In addition, we have included on the meta-analysis forest plot, percent weight for each study that is based on the effect sizes and sample sizes. The percent weights are automatically calculated and adjusted in the relative contribution of each study to the overall pooled estimate. The weight assigned to each study typically reflects the precision of the study's effect estimate, which is influenced by several factors including sample size. Larger studies usually provide more precise estimates and thus are given more weight compared to smaller studies. This may further help the interpretability of the results in the absence of funnel plot for the publication bias.

c. Item 16 (Did the review authors report any potential sources of conflict of interest, including any funding they received for conducting the review?) of AMSTAR 2.

*Response:* According to the PLOS ONE requirement, the authors declare no conflict of interest with the nature of the work described, including no funds received for this review as contained on the title page under Disclosure; line 19.

5. Finally, regarding the discussion, I felt that the explanation of the strong points of this systematic review with meta-analysis and future proposals for development was missing.

Response: Implication for future studies proposal has been added as a final paragraph to the manuscript: “While this review suggests EA is not associated with onset of new LBP or outcomes of subacute LBP, it does suggest a consistent association with outcomes of acute LBP. We have proposed potential mechanisms to explain these findings, though clearly more theoretical and empirical work is needed in this field. Future high-quality longitudinal studies with adequate sample size, clear and consistent definitions of EA and adjusting for meaning confounders in the study design and/or analysis will improve the understanding of the relative contribution of EA to the onset, and outcome, of acute and subacute LBP.”

Reviewer #2: I read the manuscript entitled "Is Educational Attainment Associated with the Onset and Outcomes of Low Back Pain? A Systematic Review and Meta-analysis" with great pleasure. The article holds excellent value for scientific and clinical audiences, adding to the evidence on the association between socioeconomic status and low back pain. It is well-written, concise, and technically sound.

I've made a few minor comments which could help to improve the manuscript further.
First, when reading the article, I needed some clarification on the specific eligibility criteria used to include articles in the systematic review. Are those the ones mentioned in the paragraph "Design", i.e., observational prospective cohort or population-level studies (not clinical trials) of patients aged >18 years with either no LBP at inception (followed to determine onset), or acute (<8 weeks) and/or sub-acute (8-12 weeks) LBP. Did you, for instance, also have specific criteria for the methods for evaluating educational attainment, the outcomes of low back pain, the language in which the articles were written, or the fact that the articles had to have been peer-reviewed?
Second, I needed help understanding the sentence with lines 199 - 201. Could you further improve this sentence?
Finally, there were some minor typos or missing words throughout the manuscript, which could be corrected.

**Response**: Your understanding is correct; we have stated in the inclusion criteria that only observational prospective studies (not clinical trials) are included, as reported in lines 134 - 135.

Educational attainment: The specific method for assessing educational attainment was stated in lines 174 -178 “Where possible, the minimum data extracted were related to a 12-year cut-point for EA as representing the threshold between secondary (up to year 12) and post-secondary (beyond year 12) EA in most countries. Where data were not presented with adequate detail, EA was sorted into meaningful order based on the manner reported in the studies (e.g., low vs high).”

Outcome of Back Pain: Line 181 – 187: “Outcomes were limited to those broadly categorized as either pain (e.g., presence/absence of LBP or pain intensity) or disability (e.g., return to work or score on a standardized patient-reported outcome). Where studies reported “recovery” as an outcome those operationalizations were reviewed for relevance and if aligned with our purpose the verbatim definition was extracted and assigned to the most relevant outcome category (e.g., pain, disability, or both)”.

Language in which the articles were written: In line 148 under the search strategy, we state that the databases were searched without restriction to the language of publication. However, we have also now included an allusion to the English-language focus as a potential limitation to interpretation in the Limitations section of the paper: “We did not include studies published in a language other than English or without a formal English translation available, raising the risk that we have missed data from work published in non-English journals that may influence our results.”

Peered reviewed: In this study, we have attempted to include not just peer-reviewed articles. Lines 148 – 149 stated, “A grey literature search of unpublished studies was conducted in Researchsquare”.

Line 199 – 201: If I got this correct, lines 199 to 201 state that “Heterogeneity in effects was assessed using both the Chi-squared test (p-value) and the I-squared statistic. I-squared <30% was deemed low heterogeneity, 30-60% as moderate, 61-75% as substantial, and 76-100% as considerable heterogeneity.”

6. PLOS authors have the option to publish the peer review history of their article ([what does this mean?](https://journals.plos.org/plosone/s/editorial-and-peer-review-process#loc-peer-review-history)). If published, this will include your full peer review and any attached files.


If you choose “no”, your identity will remain anonymous, but your review may still be made public.


**Do you want your identity to be public for this peer review?** For information about this choice, including consent withdrawal, please see our [Privacy Policy](https://www.plos.org/privacy-policy).

Reviewer #1: **Yes:** CARLOS AUGUSTO FERREIRA DE ANDRADE

Reviewer #2: No

**Response**: We sincerely thank the reviewers for their thoughtful comments and suggestions.

On behalf of the Authors

Aliyu Lawan, PhD.

Reference

Lau, J., Ioannidis, J. P. A., Terrin, N., Schmid, C. H., & Olkin, I. (2006). The case of the misleading funnel plot. *BMJ (Clinical Research Ed.)*, *333*(7568), 597–600. https://doi.org/10.1136/bmj.333.7568.597
